# Empowering hypoxia to convert cold tumors into hot tumors for breast cancer immunotherapy

**DOI:** 10.1038/s41420-025-02682-8

**Published:** 2025-08-14

**Authors:** Lu Liu, Danping Wu, Zhiwen Qian, Ying Jiang, Yilan You, YiDa Wang, Feng Zhang, Xin Ning, Jie Mei, Jabed Iqbal, Yanfang Gu, Yan Zhang

**Affiliations:** 1 Department of Oncology, Wuxi Maternity and Child Health Care Hospital, Women’s Hospital of Jiangnan University, Jiangnan University, Wuxi, China; 2 Department of Oncology, Wuxi Maternity and Child Health Care Hospital, Wuxi Medical Center, Nanjing Medical University, Wuxi, China; 3 The First College of Clinical Medicine, Nanjing Medical University, Nanjing, Jiangsu China; 4 Department of Anatomical Pathology, Singapore General Hospital, Singapore, Singapore

**Keywords:** Cancer immunotherapy, Immune evasion

## Abstract

Breast cancer remains the most common cancer among women globally and a leading cause of cancer-related death. Despite the promise of immunotherapy for triple-negative breast cancer (TNBC), its overall effectiveness is hindered by the cold tumor microenvironment (TME), characterized by sparse immune cell infiltration. This review explores the pivotal role of hypoxia in shaping the breast cancer TME and its influence on immunotherapy efficacy. As a defining feature of most solid tumors, including breast cancer, hypoxia drives aggressive tumor behavior, metastasis, and treatment resistance. The hypoxic TME promotes immune evasion and maintains the cold tumor phenotype. Targeting hypoxia offers a potential strategy for transforming cold breast tumors into hot tumors that respond more effectively to immunotherapy. This review consolidates existing insights into the interplay between hypoxia, tumor immunophenotypes, and immunotherapy in breast cancer. By analyzing the mechanisms through which hypoxia modulates the TME and immune response, it proposes innovative strategies to enhance immunotherapy outcomes. This comprehensive analysis lays the groundwork for developing more effective combination therapies to improve breast cancer prognosis.

## Facts

- Hypoxia is a common hallmark of breast cancer, intricately associated with disease progression and immune therapy responses through its modulation of various biological pathways.
- Breast cancer can be categorized into immunologically hot tumors, which exhibit strong responses to immunotherapy, and cold tumors, which show minimal responsiveness, based on the presence and distribution of immune components within the tumor microenvironment.
- Alleviating hypoxia can influence multiple signaling pathways, potentially transforming cold breast tumors into hot tumors, thereby improving their susceptibility to immunotherapy.
- Several strategies to alleviate hypoxia are available, each carrying substantial clinical significance.

## Open questions

- Are there additional pathways through which hypoxia influences breast cancer progression and response to immunotherapy?
- Is there a more comprehensive and detailed classification of hot and cold tumors in breast cancer?
- To what extent does alleviating hypoxia enhance the efficacy of immunotherapy in cold tumors?
- What is the potential of strategies to alleviate hypoxia in the clinical application of immunotherapy?

## Introduction

Breast cancer is the most prevalent malignancy among women worldwide [1]. Tumor hypoxia is a common hallmark of breast cancer, strongly correlated with increased metastatic risk and higher patient mortality [2]. Under low oxygen conditions, hypoxia-inducible factor (HIF) signaling is activated, triggering various transcriptomic alterations [3]. Hypoxia compromises anti-tumor immunity by promoting the pro-tumor M2 phenotype, fostering regulatory T cell accumulation within the tumor, and activating adenosine receptors [4]. Elevating oxygen levels in the TME has been shown to enhance the infiltration of both innate and adaptive anti-tumor immune cells, thereby boosting immunotherapy efficacy [5]. Currently, breast cancer treatment is entering the immunotherapy era, which often depends on the interaction between immune cells and the TME [6]. Immunotherapy remains challenging for breast cancer due to its classification as an immunogenic cold tumor [7]. A promising and innovative immunotherapy strategy involves converting cold tumors into hot tumors, enhancing treatment response. Understanding the relationship between hypoxia and breast cancer, distinguishing immune-hot from immune-cold tumors, and exploring effective strategies to convert cold tumors into hot ones, reveals a critical pathway for improving therapeutic outcomes. By reversing tumor hypoxia, it is possible to transform cold breast cancer tumors into immune-hot tumors, offering novel approaches for clinical immunotherapy in breast cancer.

## Mechanisms of hypoxia in cancer

**Fig. 1:**
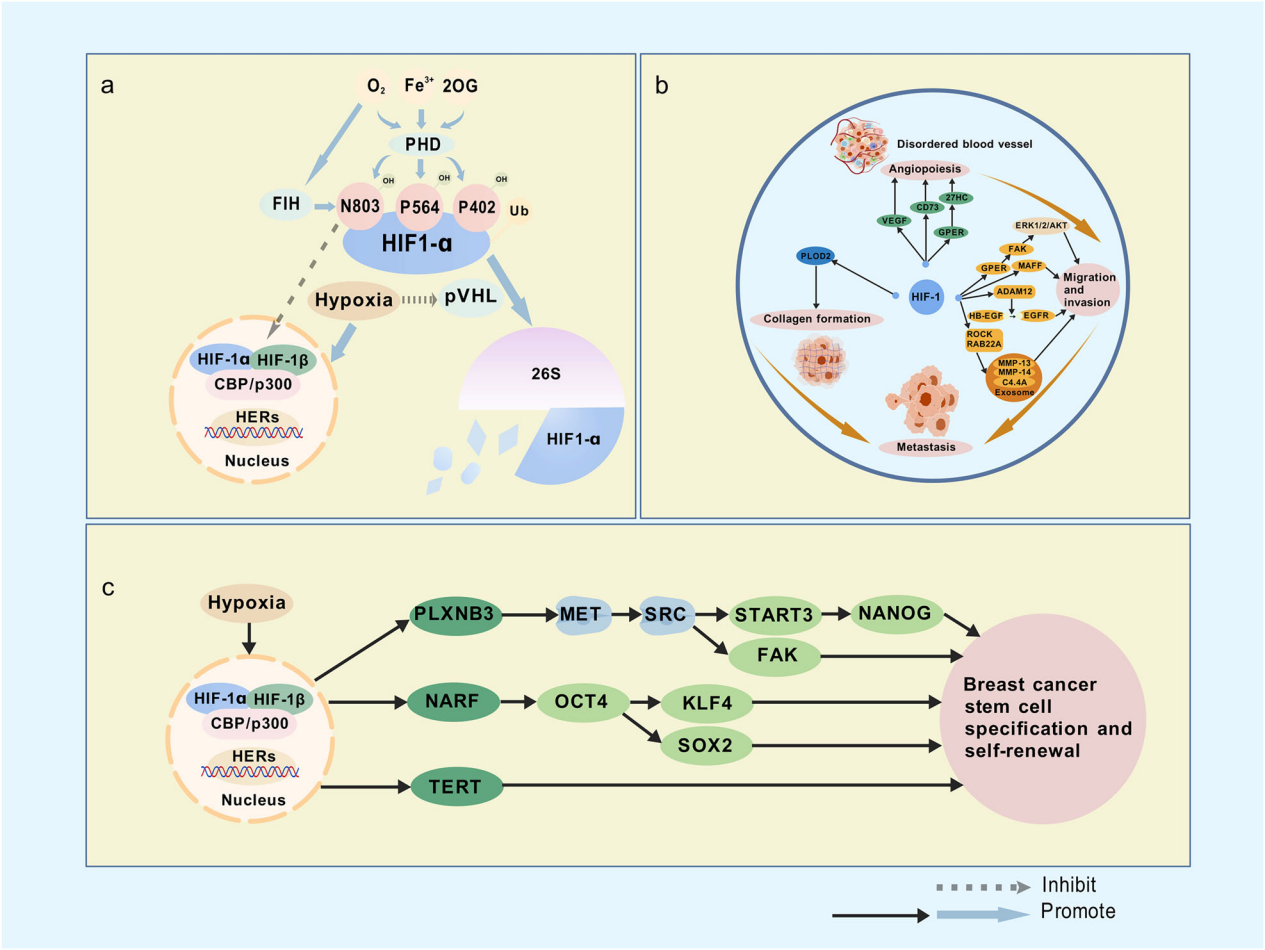
Mechanisms by which hypoxia regulates HIF-1α *via* PDH and promotes breast cancer metastasis and BCSC maintenance. **a** Under normoxic conditions, HIF-1α is hydroxylated at P402, P564, and N803 by PHDs and FIH in an oxygen-, iron-, and 2-oxoglutarate-dependent manner. These modifications facilitate HIF-1α polyubiquitination and degradation through the pVHL-26S proteasome pathway, and prevent its interaction with the transcriptional coactivators CBP/p300, suppressing its transcriptional activity. Under hypoxic conditions, hydroxylation is inhibited, resulting in HIF-1α stabilization, nuclear translocation, and dimerization with HIF-1β. The HIF-1 complex then binds to HREs in target gene promoters, initiating the transcription of hypoxia-responsive genes. **b** Hypoxia-induced activation of HIF-1 upregulates GPER, leading to VEGF expression and angiogenesis. GPER also interacts with 27HC, activating FAK and triggering downstream ERK1/2 and AKT signaling, which enhances cancer cell migration and invasion. HIF-1 further increases CD73 expression, promoting angiogenesis and metastasis through non-enzymatic functions. Additionally, HIF-1 upregulates ADAM12, which cleaves membrane-bound HB-EGF, releasing its extracellular domain to activate EGFR signaling and enhance tumor cell invasiveness. HIF-1 also induces PLOD2, facilitating collagen biosynthesis and ECM remodeling, and activates MAFF, which further supports tumor invasion. Moreover, HIF-1 stimulates the expression of RAB22A and ROCK, promoting exosome release. These exosomes contain ECM-degrading proteins such as MMP-13, MMP-14, and C4.4A, which enhance cell invasion and metastatic progression. Collectively, these pathways form a hypoxia-driven regulatory network that accelerates breast cancer progression. **c** Under hypoxic conditions, HIF-1 transcriptionally activates PLXNB3, NARF, and TERT. PLXNB3 activates MET, which signals through SRC to STAT3, inducing NANOG expression and promoting a BCSC–like phenotype. SRC also activates FAK, a key regulator of BCSCs. NARF acts as a co-activator of OCT4, enhancing the transcription of KLF4, NANOG, and SOX2, all critical for BCSC self-renewal. TERT, SOX2, and KLF4 further support stemness maintenance.

Hypoxia induces profound proteomic alterations through various hypoxia response transcription factors [8]. HIF is the principal regulator of this response. HIF consists of three oxygen-labile subunits—HIF-1α, HIF-2α, and HIF-3α—alongside the constitutively expressed HIF-1β subunit (also known as ARNT), with HIF-1α and HIF-2α being the most extensively studied [9]. While HIF-1α is ubiquitously expressed in hypoxic tissues, whereas HIF-2α is detected in a more restricted found primarily in vascular endothelial cells and macrophages, and often expressed under both hypoxic and normoxic conditions. Notably, HIF-1α primarily regulates genes involved in anaerobic glycolysis and cell death, while HIF-2α controls genes related to erythropoietin (EPO) synthesis and tumor stemness or pluripotency. HIF-1 is regarded as the central mediator of cellular responses to hypoxia [10]. It is regulated by various factors and, through a series of biochemical events, binds to hypoxia response elements (HREs) activating the transcription of numerous of target genes, that modulate the cell’s adaptation to low oxygen availability [11] (Fig. 1a). HIF-1α undergoes hydroxylation at two specific proline residues (P402 and P564) and one specific asparagine residue (N803) by prolyl hydroxylase domain proteins(PHDs), which are oxygen-, iron-, and 2-oxoglutarate(2OG)-dependent dioxygenases. This modification triggers polyubiquitination of HIF-1α, marking it for degradation by the Von Hippel-Lindau tumor suppressor gene (pVHL) for degradation via the 26S proteasome. This degradation process reduces HIF-1α‘s transactivation activity, ensuring its transcriptional functions are activated only under hypoxic conditions. The Hypoxia-Inducible Factor Inhibitor (FIH) is an oxygen-dependent enzyme that hydroxylates asparagine residues such as Asn-803, on the HIF-1α subunit, thereby disrupting its interaction with CBP/p300 and inhibiting its transcriptional activity. Under hypoxic conditions, the loss of pVHL function results in the accumulation of HIF-1α. The stabilized HIF-1α subunits then translocate to the nucleus, where they form a heterodimer with HIF-1β, bind to CBP/p300 and activate HREs in target gene regulatory regions. In addition to HIF, several other transcription factors play key roles in the cellular response to hypoxia.

## Hypoxia promotes angiogenesis, metastasis, and invasion in breast cancer

Hypoxia promotes aberrant neovascularization, enhances the migratory and invasive capacities of breast cancer cells, and stimulates collagen deposition in the extracellular matrix(ECM), ultimately facilitating breast cancer metastasis (Fig. 1b). Hypoxia upregulates GPER in breast cancer through HIF-1, thereby activating VEGF expression and angiogenesis [12]. In TNBC, GPER activation is linked to focal adhesion kinase (FAK) activation, triggering ERK1/2/AKT signaling, which promotes metastatic traits such as migration and invasion. GPER also interacts with 27-hydroxycholesterol(27HC), further inducing tumor angiogenesis [13]. HIF drives angiogenesis by inducing the secretion of pro-angiogenic growth factors from both tumor and stromal cells [14]. The newly formed vasculature is disordered and permeable, which facilitates tumor cell invasion and metastasis. Through HIF-dependent upregulation of a disintegrin and metalloprotease 12 (ADAM12), hypoxia cleaves the extracellular domain of the membrane-bound heparin-binding EGF-like growth factor (HB-EGF). Once released, this extracellular domain binds to the epidermal growth factor receptor (EGFR), activating signaling pathways that enhance the migratory and invasive potential of breast cancer cells, leading to metastasis [15]. HIF-1 induces PLOD2, which encodes an enzyme critical for collagen biosynthesis, a major ECM component. This process enhances ECM remodeling, promoting tumor invasion. Additionally, HIF-1α recruits TET1 to demethylate the ATF3 promoter, activating ATF3 transcription, which drives alternative splicing of P4HA1 to generate the P4HA1-9a isoform that promotes collagen deposition in the ECM and increases tumor cell invasiveness [16]. HIF-1 also upregulates CD73, further contributing to breast cancer angiogenesis and metastasis. Another target of HIF-1, MAFF, is induced under hypoxia, enhancing tumor cell invasiveness [17]. Furthermore, HIF-1α can promote exosome release by transactivating RAB22A, which facilitates intercellular communication within the tumor microenvironment. Hypoxia also induces RHO-associated protein kinase (ROCK), which promotes exosome biogenesis across various tumor cells. These exosomes contain ECM-degrading proteins such as MMP-13, MMP-14, and C4.4A [18]. Exosomal MMP-13 significantly upregulates vimentin expression and downregulates E-cadherin in recipient cells, thus driving cell invasion both in vitro and in vivo [18].

## Hypoxia promotes the maintenance of breast cancer stem cells

Hypoxia exposure enriches the population of breast cancer stem cells (BCSCs) [19]. HIF-1-dependent transcriptional activation of PLXNB3, NARF, and TERT, under hypoxic conditions drives the expansion and self-renewal of BCSCs (Fig. 1c). PLXNB3 interacts with and activates the MET receptor tyrosine kinase, which then signals through the non-receptor tyrosine kinase SRC [20]. SRC subsequently activates FAK, a critical regulator of BCSC specification as well as breast cancer invasion and metastasis. Additionally, SRC signals to STAT3, which in turn induces NANOG transcription. Hypoxia-induced expression of NARF is HIF-1α-dependent but independent of HIF-2α. NARF acts as a coactivator of OCT4, promoting the transcription of KLF4, NANOG, and SOX2 [21]. Hypoxia also drives TERT expression through HIF-1. NANOG is recruited to the HIF-1 binding site within the TERT gene, and NANOG knockdown disrupts the binding of hypoxia-induced HIF-1α and HIF-1β to the TERT promoter. Chronic hypoxia remodels stemness remodeling in breast cancer cells by upregulating HIF-2α, which in turn increases SOD2 expression to reduce mitochondrial reactive oxygen species (mtROS) levels. This reduction leads to downstream biological effects, including GRP78-UPRER activation and further stemness remodeling [22].

## Hypoxia promotes metabolic reprogramming in breast cancer

**Fig. 2:**
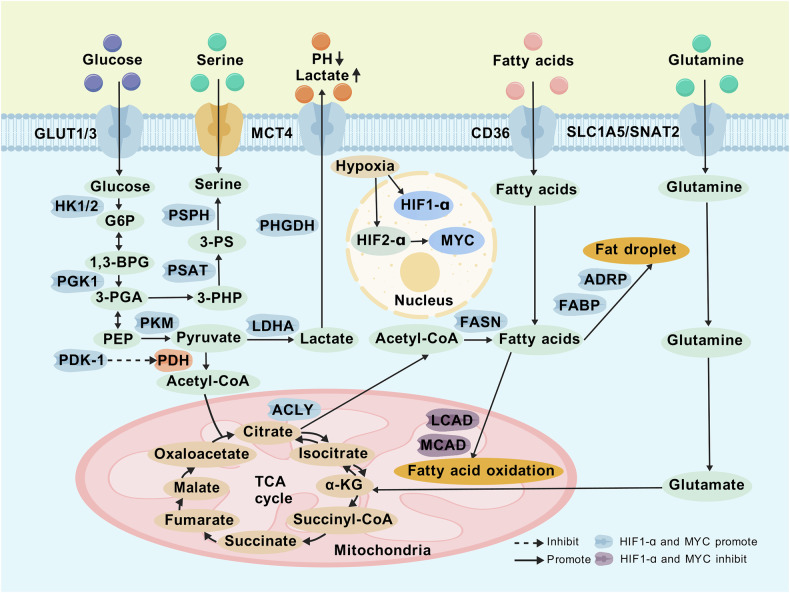
Mechanisms by which hypoxia promotes metabolic reprogramming in breast cancer. In hypoxic conditions, the transcription factors HIF-1α and MYC are activated in the nucleus and drive metabolic reprogramming of cancer cells by regulating various related genes involved in glucose metabolism, amino acid metabolism, and fatty acid metabolism. This series of reactions, along with the production of metabolic by-products, supports cancer cell survival, proliferation, and tumor progression.

Cancer cell metabolism encompasses glucose, amino acid, and fatty acid metabolic pathways. Under hypoxic conditions, these pathways undergo reprogramming to support cell survival and proliferation, thereby facilitating tumor progression (Fig. 2). In response to low oxygen, the HIF-1α transcription factor drives metabolic reprogramming by regulating multiple glycolysis-related genes, such as GLUT1, GLUT3, PKM2 and LDHA, enhancing glycolysis to meet the high demands of rapid proliferation. HIF-1α also inhibits pyruvate dehydrogenase (PDH) activity by activating pyruvate dehydrogenase kinase 1 (PDK-1), reducing the flow of pyruvate into the TCA cycle and promoting lactate production. Beyond HIF-1α, hypoxia can activate the oncogenic transcription factor MYC, which further enhances lactate production [23]. While HIF-1α, predominates in acute hypoxia, HIF-2α is more active in chronic hypoxia and exhibits cell-type-specific expression. In the TME, HIF-2α expression can be elevated, leading to overexpression of c-MYC. MBP-1, a negative regulator of c-MYC, is inhibited under hypoxic conditions, disrupting the MBP-1/c-MYC promoter interaction and reducing the negative regulation of c-MYC [24]. MYC influences various cellular processes and modulates the host immune response and TME [25]. Both HIF-1α and MYC share common targets in glycolysis [26], as well as in amino acid and lipid metabolism. Lactate is exported from cells *via* the MCT4 transporter, acidifying the TME and promoting metastasis, angiogenesis, and immunosuppression, all of which are associated with poor clinical prognosis [27]. HIF-1α induces the expression of fatty acid–binding proteins (FABP3 and FABP7) and ADRP, key factors required for lipid droplet formation, thereby promoting lipid droplet accumulation and uptake. Furthermore, HIF-1α suppresses mitochondrial fatty acid oxidation by inhibiting medium-chain acyl-CoA dehydrogenase (MCAD) and long-chain acyl-CoA dehydrogenase (LCAD), reducing ROS production and blocking the PTEN pathway, which ultimately favors tumor cell proliferation. ATP citrate lyase (ACLY), a target of HIF-1α, is upregulated in hypoxic tumor cells [28]. ACLY plays a pivotal role in fatty acid synthesis and acetyl-CoA generation. Hypoxia also significantly affects the metabolism of two non-essential amino acids, glutamine and serine, in cancer cells. Glutamine serves as a key substrate, providing both carbon and nitrogen. Its uptake is mediated by glutamine transporters (SLC1A5 and SNAT2), which are upregulated in a HIF-1α–dependent manner under hypoxic conditions [29]. Glutamine is converted into glutamate, which is then further metabolized by glutamate dehydrogenase or transaminases to form α-ketoglutarate (αKG). αKG can enter the TCA cycle or be converted to citrate *via* isocitrate dehydrogenase (IDH) [30]. Under hypoxia, HIF-1α orchestrates metabolic pathways to enhance glutamine-dependent reductive carboxylation, promoting tumor cell growth and proliferation [31]. Serine is involved in synthesizing other amino acids (e.g., glycine, cysteine) and phospholipids (e.g., phosphatidylserine), and it contributes one-carbon units to the folate cycle, making it indispensable for cancer cell growth. Under hypoxic conditions, serine synthesis is driven by three enzymes—phosphoglycerate dehydrogenase (PHGDH), phosphoserine aminotransferase (PSAT), and phosphoserine phosphatase (PSPH)—whose expression is induced by HIF-1α and is crucial for cancer cell proliferation.

## Hypoxia promotes immune evasion in breast cancer

**Fig. 3:**
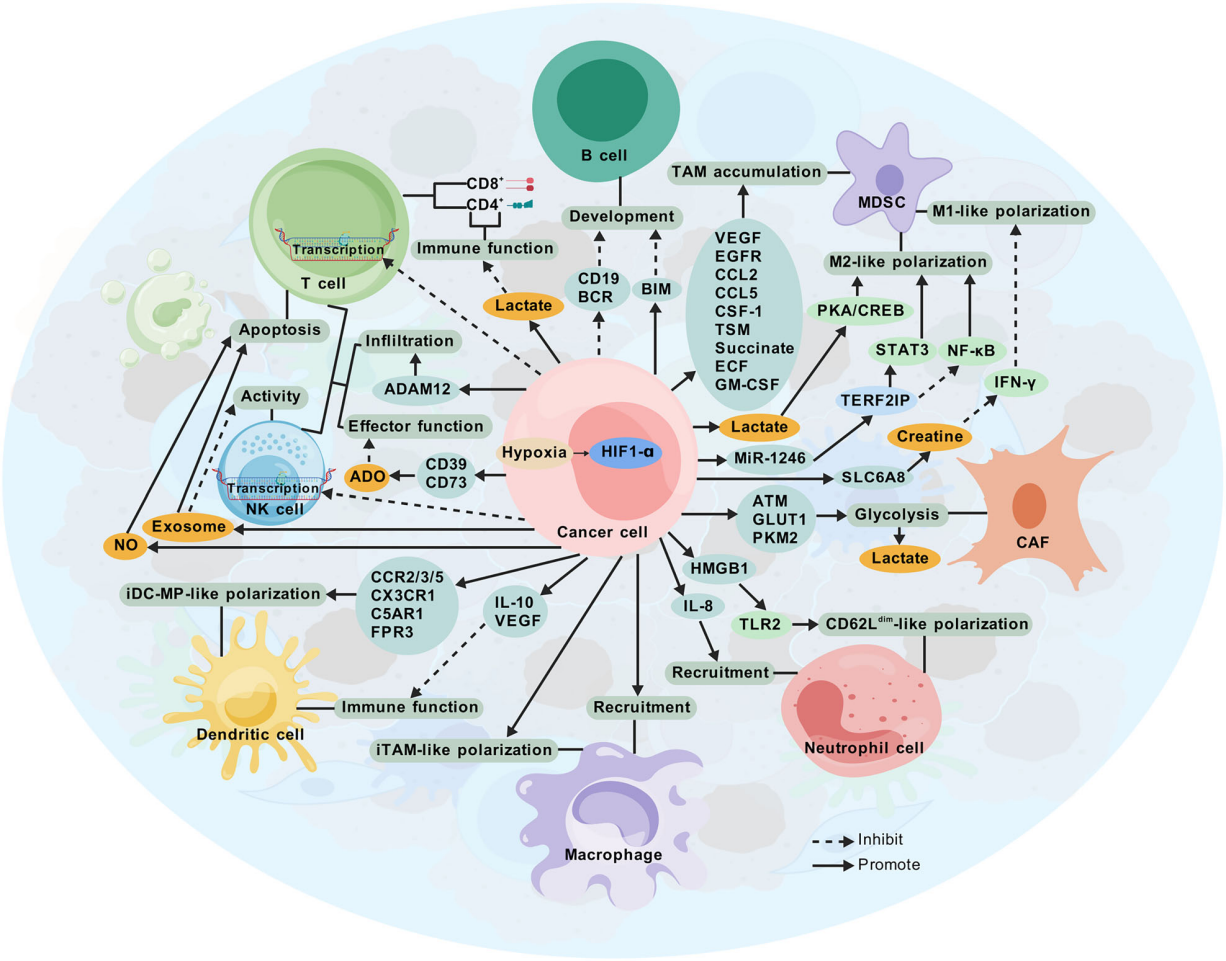
Effects of hypoxia on immune cells in breast cancer and its mechanisms. In breast cancer, hypoxia regulates immune function through multiple mechanisms. HIF-1α transcriptionally activates various genes, produces diverse metabolic by-products, and influences numerous signaling pathways, all of which differentially impact the growth, development, differentiation, and functional formation of immune cells, including T cells, NK cells, B cells, MDSCs, CAFs, neutrophils, macrophages, and DCs. Ultimately, hypoxia suppresses immune function in breast cancer, promoting immune evasion and contributing to tumor progression.

Hypoxia promotes immune evasion in breast cancer by modulating immune cells within the TME. Through HIF-1α, hypoxia affects various genes, cytokines, chemokines, signaling pathways, and metabolic products through HIF-1α, further influencing immune cells and ultimately leading to immune evasion in breast cancer (Fig. 3).

## Hypoxia induces dysfunction in T and NK effector cells

Hypoxia-induced HIF-1α-dependent epigenetic reprogramming enhances the transcriptional repression of effector genes in human T cells and natural killer (NK) cells [32]. Additionally, hypoxia can also reduce the infiltration of CD8+ T cells and NK cells by upregulating ADAM12 [33]. Accumulation of lactate due to hypoxia inhibits the function of CD8+ and CD4+ effector T cells, while promoting helper T-cell differentiation and increasing interferon-γ (IFN-γ) production [34]. Hypoxia also upregulates CD39 and CD73 *via* HIF-1α [35], leading to the sequential conversion of ATP into extracellular adenosine (ADO) [36]. ADO binds to the A2A adenosine receptor (A2AR), inhibiting interleukin-2 (IL-2) production and impairing T-cell development and proliferation [37]. Additionally, ADO suppresses the effector functions of NK cells and dendritic cells (DCs) and promotes the recruitment and polarization of myeloid-derived suppressor cells (MDSCs) and regulatory T cells (Tregs), thus dampening antitumor immunity [38]. Hypoxia also promotes both endocytosis and exosome release, with tumor-derived exosomes inducing T-cell apoptosis and reducing NK cell activity. In the hypoxic TME, HIF-1α is significantly upregulated, enhancing the expression of inducible nitric oxide (NO) synthase and arginase-1 (ARG-1) [39]. NO can induce T-cell apoptosis and nitrosylate chemokines and the T-cell receptor (TCR), thereby inhibiting T-cell migration and cytotoxicity against tumor cells. These molecules also suppress cytokine production, such as IL-2, which is essential for T-cell antitumor function.

## Hypoxia affects B cells

Chronic hyperactivity of HIF-1α under hypoxia reduces the surface abundance of B-cell receptor (BCR), CD19, and the B-cell activator receptor, while increasing the expression of the proapoptotic protein BIM, thus hindering normal B-cell development [40]. HIF-1α interacts with CXC chemokine receptor 4 (CXCR4) to enhance B-cell survival. Moreover, hypoxia-induced activation of HIF-1α triggers autocrine TGF-β signaling, facilitating myofibroblast activation, CXCL13 induction, B-lymphocyte recruitment, and MYC secretion—mechanisms that promote B-cell proliferation and survival [40].

## Hypoxia influences macrophages

Hypoxia induces the production of various chemoattractants in the tumor stroma and hypoxic regions, including VEGF, EGFR, CCL2, CCL5, CSF-1, oncostatin M, succinate, eosinophil chemotactic factor, and GM-CSF [41]. These factors recruit tumor-associated macrophages (TAMs) and promote their M2-like polarization. Breast cancer cells with ectopic Zeb1 expression produce lactate in the acidic tumor environment, activating the PKA/CREB signaling pathway, which selectively promotes M2 macrophage polarization. Hypoxia also regulates miRNA production in tumor-derived exosomes through HIF. MiRNAs, a class of regulatory noncoding RNAs (ncRNAs) are found in various exosomes [42]. MiR-1246 targets telomere repeat-binding factor 2-interacting protein (TERF2IP), significantly promoting M2 macrophage polarization by activating STAT3 and inhibiting the NF-κB pathway, leading to enhanced tumor proliferation, migration, and invasion. Moreover, exosomes enriched with miR-1246, from hypoxic tumor cells can be transferred to normoxic tumor cells, inducing increased motility and invasiveness. Hypoxia induces HIF-dependent upregulation of ADAM12, expressed by slowly cycling PDGFRα mesenchymal perivascular cells, which stimulates angiogenesis and immunosuppression by promoting TAM exocytosis and polarization. Additionally, hypoxia upregulates SLC6A8 expression, causing creatine accumulation, which critically supports breast cancer progression [43]. Creatine inhibits IFN-γ-dependent proximal signaling in macrophages in an energy-independent manner, attenuating M(IFN-γ) polarization [44].

## Hypoxia impacts cancer-associated fibroblasts

Cancer-associated fibroblasts (CAFs) are present in various cancers, including breast cancer [45]. Hypoxia drives glycolytic activity in CAFs *via* ATM oxidation, GLUT1 phosphorylation, and overexpression of PKM2 [46]. Lactate produced by CAFs enhances breast cancer cell invasion by activating the TGFβ1/p38 MAPK/MMP2/9 axis and driving mitochondrial OXPHOS [46]. G-protein–coupled estrogen receptor (GPER), a transcriptional target of HIF-1α, mediates a feedforward loop involving IL-1β–induced CAF activation and IL1R1 expression in breast cancer cells, jointly regulating target genes and relevant biological processes [47].

## Hypoxia affects neutrophils

Under hypoxic conditions, neutrophils increase mitochondrial membrane potential and generate mitochondrial reactive oxygen species (mtROS) through a mitochondrial pathway involving the 3-phosphoglycerate shuttle, which further stabilizes HIF-1α [48]. HIF-1α promotes the release of HMGB1 from tumor cells [49]. CD62L, a member of the selectin family, plays a pivotal role in neutrophil movement. The adhesion of CD62L^dim^-neutrophils is weaker than that of CD62L^hi^-neutrophils. Through TLR2 signaling, HMGB1 induces neutrophils to adopt a CD62L^dim^ phenotype, promoting metastatic formation [50]. Hypoxia-induced interleukin-8 (IL-8) is another critical factor for recruiting neutrophils within tumors.

## Hypoxia shapes myeloid-derived suppressor cells

HIF-1 also drives immune evasion and promotes the recruitment of MDSCs. In TNBC, MDSCs regulate both tumor-killing and immunosuppressive cells by secreting cytokines and inhibiting antitumor immunity. MDSCs secrete CCL5, which binds CCR5 on TNBC cells, promoting further MDSC recruitment [51]. Hypoxia facilitates the differentiation of MDSCs into immunosuppressive TAMs, intensifying immune suppression in the TME [52].

## Hypoxia influences dendritic cells

DCs are professional antigen-presenting cells (APCs) that bridge innate and adaptive immunity. HIF-1, a heterodimeric DNA-binding transcription factor, negatively regulates DC function under low-oxygen conditions [53]. HIF-α mediates the upregulation of various chemokine receptors (CC-chemokine receptor 2/3/5, CX3C chemokine receptor 1, C5a receptor gene 1, and formyl peptide receptor 3), polarizing immature DCs (iDCs) into a migratory phenotype and enhancing their motility *via* PI3K/AKT signaling [54]. HIF-α-mediated secretion of VEGF and Interleukin-10 (IL-10) further inhibits DC function [55].

## Cold tumors and hot tumors in breast cancer

Tumors can be classified into three major immune phenotypes based on the arrangement and presence of cytotoxic immune cells within the TME: immune-inflamed, immune-excluded, and immune-desert [56]. Immune-inflamed tumors, or hot tumors, are characterized by significant T-cell infiltration, enhanced IFN-γ signaling, increased programmed death-ligand 1 (PD-L1) expression, and a high tumor mutational burden (TMB) [57]. In contrast, immune-excluded and immune-desert tumors, or cold tumors, display a lower mutational burden, reduced expression of major histocompatibility complex (MHC) class I molecules, and low PD-L1 levels. These tumors also harbor immunosuppressive cell populations, such as TAMs, Tregs, and MDSCs [58], and exhibit a marked scarcity of CD8+ T lymphocytes in and around the tumor [59]. Various factors within the tumor can impair the ability of immune cells in the TME to eliminate malignant cells or reprogram these cells to support tumorigenesis. For example, APCs like DCs and macrophages, can be skewed toward immunosuppressive phenotypes by TAM polarization, MDSC expansion, or inhibition of DC maturation, thereby creating an immunosuppressive TME that hinders the activation and function of antitumor CD8+ T cells [60]. Both CD8+ T cells and NK cells play essential roles in immunosurveillance to control tumor growth and metastasis, representing the adaptive and innate arms of antitumor immunity, respectively [61]. Consequently, CTLs and NK cells serve as key indicators to distinguish hot tumors from cold tumors. NK cells, which coexpress PD-1 and PD-L1, are critical for the therapeutic efficacy of PD-1/PD-L1 checkpoint blockade in various murine models. Depletion of NK cells diminishes the antitumor effects of PD-L1 inhibition, thereby promoting tumor progression [62].

## Mechanisms for converting cold tumors into hot tumors

### Improving T-cell infiltration

#### Principles of T-cell infiltration

**Fig. 4:**
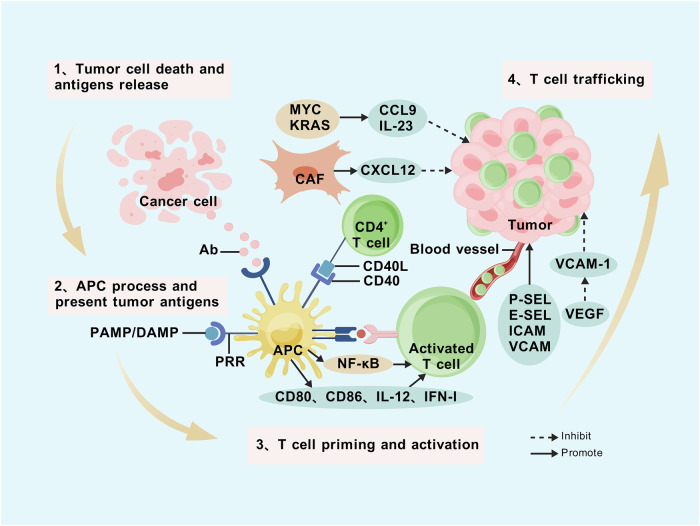
Steps and influencing factors in driving T cells into the TME. The process of driving T cells into the TME involves four key steps: tumor cell death and antigen release, processing and presentation of tumor antigens by APCs, the interaction between APCs and T cells leading to T-cell priming and activation, and the migration of activated T cells from lymph nodes to the tumor site *via* the bloodstream. APCs expressing PRRs are directly activated by PAMPs or DAMPs, thereby acquiring the capacity to initiate T-cell responses. The binding of PRRs to DCs within APCs triggers NF-κB activation, promoting cross-priming. A key step in APC activation and subsequent CD8+ T-cell priming is the stimulation of CD40 on APCs by CD40L expressed on helper CD4+ T cells. This stimulation enhances the expression of CD80 and CD86, increases cytokine production (including IL-12 and IFN-I), and promotes T-cell activation. Co-activation of KRAS and MYC leads to the production of CCL9 and IL-23, mediating stromal reprogramming, promoting angiogenesis, and excluding T cells, B cells, and NK cells from the tumor. CAFs secrete CXCL12, which misdirects CTLs into the peritumoral stroma, preventing their infiltration into the tumor. The recruitment of CD8+ T cells to the tumor requires endothelial adhesion molecules, such as P-selectin, E-selectin, ICAMs, and VCAMs. VEGF downregulates the expression of key endothelial adhesion molecules, like VCAM-1, thus hindering T-cell migration into the TME.

The process of T cell infiltration into the TME is a multi-step, progressive event (Fig. 4). It begins with tumor-cell death and antigen release, followed by the processing and presentation of tumor antigens by APCs. The interactions between APCs and T cells then lead to T cell priming and activation [63]. Activated tumor-specific T cells typically leave the lymph nodes and travel *via* the bloodstream to the tumor site.

## Mechanisms underlying deficits in T-cell infiltration

T-cell infiltration can be hindered by several factors, including insufficient tumor antigens, APC dysfunction, impaired T-cell activation, or defective T-cell homing to the tumor bed. Recognition of neoantigens can enhance both T-cell priming and infiltration. Naive T cells require interaction with activated APCs in the presence of appropriate “danger signals” to initiate T-cell responses. APCs equipped with pattern-recognition receptors (PRRs) are activated by pathogen-associated molecular patterns (PAMPs) or damage-associated molecular patterns (DAMPs), allowing them to prime T cells. When DCs detect PAMPs or DAMPs, they trigger NF-κB activation, upregulate costimulatory molecules, produce cytokines, and initiate cross-priming. However, insufficient or low-level DAMP generation may result in incomplete DC maturation and the production of immunosuppressive factors such as TGF-β, leading to inadequate CD4+ T-cell support. Another critical step in APC activation occurs when CD40 on APCs is stimulated by CD40L expressed on helper CD4+ T cells, driving the priming of CD8+ T-cells. CD40 engagement on DCs modulates the expression of costimulatory molecules,such as CD80 and CD86, enhances the production of key cytokines (including IL-12 and type I IFNs), and facilitates cross-priming of exogenous antigens. Impaired APC activation or lack of costimulation can contribute to diminished CD8+ T-cell responses. The mechanisms regulating T-cell homing to tumor sites are more complex. Oncogenic pathways, such as WNT/β-catenin signaling and BATF3-DC deficiency, mediate T-cell exclusion and immune evasion by tumor cells [64]. PTEN loss upregulates the PI3K/AKT pathway, suppressing T-cell priming and T-cell-mediated antitumor immunity [65]. Oncogenic RAS activates several signaling pathways (e.g., MAPK and PI3K) that promote tumorigenesis [66]. Coactivation of KRAS and MYC enhances the secretion of tumor-derived CCL9 and IL-23, driving stromal reprogramming, promoting angiogenesis, and excluding tumor-infiltrating T, B, and NK cells [67]. CAF-produced CXCL12 may misdirect CTLs to the peritumoral stroma, preventing their penetration into the tumor. Additionally, the vascular trafficking of CD8+ T cells into the tumor is dependent on endothelial adhesion molecules. Vascular endothelial growth factor (VEGF) reduces the endothelial expression of adhesion molecules essential for T-cell migration, thus hindering T-cell infiltration into the TME.

## Specific strategies to improve T-cell infiltration

### Promoting T-cell priming

T-cell priming begins with antigen presentation by activated DCs in secondary lymphoid organs. Immunologic adjuvants can stimulate DC maturation, enhancing the expression of MHC-II, CD40, and CD86, thereby improving the generation of tumor-specific CD8+ T cells and promoting tumor suppression [68]. Oncolytic viruses (OVs) induce immunogenic cell death (ICD) upon tumor cell lysis, resulting in the release of tumor-associated antigens (TAAs), PAMPs, and DAMPs [69]. OVs also enhance DC function by stimulating type I IFN production, increasing the release of CXCL9 and CXCL10, and upregulating adhesion molecules, which are critical for T-cell trafficking. Additionally, the enzymatic degradation of the ECM by OVs aids in breaking down physical barriers to T-cell infiltration [70]. Chemotherapy, radiotherapy, or local hyperthermic ablation can also moderately facilitate T-cell priming.

## Expanding antigen-specific T-cell populations

Adoptive cell therapy (ACT), including TIL therapy and engineered TCR/CAR-T cell therapies, along with therapeutic cancer vaccines, can expand the pool of antigen-specific T cells in circulation [71]. These strategies not only enhance systemic immunity but also improve T-cell trafficking to tumor sites, bridging peripheral immunity and local tumor control.

## Promoting T-cell trafficking and infiltration

Inhibiting oncogenic pathways offers another approach to enhancing T-cell infiltration. For instance, erdafitinib blocks the FGFR signaling axis, significantly impeding tumor growth while increasing T-cell infiltration. Mechanistically, FGFR blockade suppresses CAF proliferation and migration, as well as the release of vascular cell adhesion molecule 1 (VCAM-1) *via* MAPK/ERK pathway downregulation in CAFs, disrupting both physical and chemical barriers in the tumor immune microenvironment (TIME) [72]. Epigenetic modulators, anti-angiogenic treatments, TGF-β inhibitors, and CXCR4 antagonists also promote T-cell infiltration into tumors. Furthermore, nanomedicine-based immunotherapies offer tumor-targeted delivery platforms that utilize multiple mechanisms—targeting tumor cells, the surrounding stroma, or the peripheral immune system—to facilitate the conversion of cold tumors into hot tumors.

## Modulating the abundance and functionality of NK cells

NK cells, innate lymphocytes, eliminate tumor cells that lack MHC class I by recognizing stress signals, making them particularly effective against tumors with deficient antigen presentation machinery, such as MHC class I loss. Tissue-resident NK cells can also modulate the antitumor microenvironment by influencing therapeutic interventions targeting the IFN-γ/interleukin-12 (IL-12) axis [73]. The IFN-γ/IL-12 axis is pivotal for bridging innate and adaptive immune responses in cancer immunity [74]. IL-12 primarily produced by DCs in the TME, stimulates both T cells and NK cells to secrete cytokines and exert cytotoxic effects [75]. Hypoxia promotes the release of ATP and AMP, which are cleaved by ectonucleotidases (CD39 and CD73) into ADO [76]. Additionally, ADO signaling *via* the A2A receptor—the principal ADO receptor on NK cells—suppresses their effector functions [77].

## Transforming cold tumors into hot tumors in breast cancer by alleviating hypoxia

### Alleviating hypoxia to promote immune-cell infiltration

HIF-1α serves as an upstream regulator of numerous angiogenic factors, directly inducing the transcription of vascular growth factors and promoting tumor angiogenesis [78]. Hypoxia-induced factors, such as CCL28 and VEGF, not only stimulate angiogenesis but also influence T-cell trafficking [79]. Enhancing tumor oxygenation can downregulate the expression of angiogenic factors in tumor cells, reducing neovascularization and facilitating improved T-cell migration into the TME. This promotes better homing to the tumor bed, enhancing the infiltration of immune cells, including T cells and NK cells, and intensifying their cytotoxic responses against tumor cells. Alleviating tumor hypoxia increases intratumoral immune-cell accumulation, thereby improving immune response efficacy.

### Alleviating hypoxia to strengthen T-cell and NK-cell function

Hypoxia directly impairs T-cell function. Under low-oxygen conditions, T-cell reactivity and potency are diminished, impairing their ability to recognize and eliminate tumor cells. Research indicates that hypoxia, through the upregulation of HIFs, fosters an immunotolerant and immunosuppressive environment that hampers T-cell functionality. Reducing hypoxia alleviates the transcriptional suppression of effector genes in T cells and NK cells, leading to increased expression levels of these cells. Elevated oxygen levels enhance MHC-I expression in cancer cells, thereby improving T-cell–mediated cytotoxicity [80] and supporting NK cell anticancer activity [81]. Additionally, relieving hypoxia can reduce ADAM12 expression, promoting the infiltration of CD8+ T cells and NK cells that produce IFN-γ. Alleviating hypoxia may also reduce B-cell proliferation and survival, mitigate macrophage-mediated T-cell dysfunction, and consequently delay disease progression or enhance responses to chemotherapy and PD-1 blockade [82]. By inhibiting CD39 and CD73 ectonucleotidase expression, alleviating hypoxia diminishes the suppression of key cytokines such as IL-2, thus promoting T-cell development and proliferation. It also reduces the release of ATP and AMP, along with the conversion of AMP to ADO, lessening ADO-mediated suppression of NK cells and enhancing their effector functions. In the TME, hypoxia typically promotes the recruitment and accumulation of immunosuppressive cells, particularly Tregs and MDSCs [83], which secrete factors like IL-10 and TGF-β that suppress T-cell function and enable immune evasion. Correcting hypoxia restricts the expansion of Tregs and MDSCs, reduces immunosuppressive factors, and restores normal immune function, thereby enhancing immune responses.

## HIF-1 regulation of PD-L1 expression

HIF-1 regulates PD-L1 expression by directly binding to hypoxia-response elements in the proximal PD-L1 promoter [84]. Numerous studies have demonstrated that immune checkpoint inhibitor, such as PD-1/PD-L1 antagonists, enhance the cytolytic activity of immune cells against tumors, though their efficacy can be compromised by hypoxic conditions. By increasing intratumoral oxygen levels or reshaping the immunological milieu, the effectiveness of immune checkpoint blockade can be enhanced, thereby transforming the TME from cold to hot.

## Specific methods for alleviating hypoxia and clinical applications

Strategies to address hypoxia in breast cancer include HIF inhibitors [73], hypoxia-activated prodrugs (HAPs) [85], and improving local oxygenation to reprogram the TME [86]. Targeting hypoxia-inducible signaling, especially HIF-1α, has proven to be an effective approach [87]. However, HIF-1α inhibitors for breast cancer remain primarily in preclinical stages, highlighting the urgent need for potent, low-toxicity inhibitors with favorable druggability. These inhibitors disrupt various stages of the HIF pathway [88]. Current small-molecule HIF-1α inhibitors include the KC7F2 series, LXY6090, quercetin, arylcarboxamide derivatives, LXY6006, aminoflavones, 7-hydroxy enaminomellactone A, the DJ12 series, rotenone derivatives, PX-478 analogs, and methyl alpinumisoflavone [89, 90]. In the 1980s, the concept of targeting tumor hypoxia using hypoxia-specific cytotoxins, now known as HAPs, emerged [91]. HAPs are selectively activated in hypoxic conditions *via* endogenous cellular oxidoreductases, converting into cytotoxic agents. Enhancing local oxygenation can be achieved by increasing oxygen supply, facilitating diffusion and reducing consumption [92]. Hyperbaric oxygen (HBO) therapy, widely used in clinical settings, alleviates hypoxia in solid tumors by increasing the oxygen dissolved in the plasma [93]. Approaches that reduce ECM pressure can reopen collapsed microvasculature caused by tissue stress, thereby improving perfusion and oxygen delivery [94]. Exercise training (ExTr), defined by cumulative exercise sessions, normalizes blood vessels, improving tumor perfusion and elevating oxygenation even under resting conditions [95]. Nanomaterial-based strategies represent innovative solutions to mitigate hypoxia. Photodynamic therapy (PDT), a highly selective and minimally invasive treatment alternative to radiotherapy and chemotherapy, is widely utilized in cancer therapy [96]. However, intratumoral hypoxia often limits PDT efficacy. The administration of external *via* nanocarriers, such as perfluorocarbons (PFCs), hemoglobin (Hb), and red blood cells (RBCs), effectively relieves hypoxia and enhances PDT within tumors [97]. Elevated levels of H_2_O_2_ in the TME can act as a key signal, prompting tumors to resist further damage. Manganese dioxide (MnO_2_) nanoparticles react with endogenous H_2_O_2_ to generate dissolved oxygen in the TME. Various MnO_2_-based nanomaterials effectively convert H_2_O_2_ into O_2_, modifying the hypoxic TME [98]. Recently, agents like metformin (Met), have been explored for their ability to reverse hypoxia-driven metabolic shifts and improve the efficacy of immunotherapy and radiotherapy. As an immunometabolic regulator, Met alleviates hypoxia in certain cancers by inhibiting mitochondrial respiratory chain complex I (NADH dehydrogenase), thereby suppressing cancer cell respiration [99]. Additionally, clinical trials are increasingly investigating strategies that exploit hypoxia-induced metabolic vulnerabilities in tumors to enhance anti-tumor immunity and improve therapeutic outcomes. Glutaminase inhibitors are under clinical evaluation, particularly for TNBC, where glutamine metabolism is essential for tumor cell survival. Furthermore, lactate transport inhibitors targeting monocarboxylate transporters (MCT1/MCT4) are being tested to reduce lactate-mediated immunosuppression in the TME.

## Conclusions and perspectives

This review addresses the current challenges and critical issues in enhancing the efficacy of breast cancer immunotherapy, with a particular focus on the key factor influencing immunotherapy outcomes: the TME. By offering a detailed analysis of the TME, the review introduces the concept of the hypoxic microenvironment as a hallmark of cancer and elucidates the relationship between hypoxia and various cancers, including breast cancer. Hypoxia profoundly impacts large-scale proteomic changes through various transcription factors, influencing gene expression, cell growth, differentiation, angiogenesis, cancer stem cell maintenance, metabolic reprogramming, immune evasion, and survival responses, as well as invasion and metastasis in breast cancer. This review also provides a comparative overview of cold and hot tumors in breast cancer, based on TME characteristics and responses to immunotherapy. It also discusses potential strategies to enhance immune infiltration, such as enhancing T-cell and NK cell infiltration. Furthermore, it highlights that the series of TME alterations associated with hypoxia in breast cancer may contribute to or reflect mechanisms underlying the immunological features of cold tumors, suggesting a potential link between hypoxia and tumor immune phenotypes. Thus, enhancing hypoxia represents a viable strategy for converting cold tumors into hot tumors in breast cancer. Finally, the review summarizes various methods for enhancing hypoxia, including HIF inhibitors, HAPs, and strategies for improving in situ oxygenation. It suggests that improving hypoxia could transform cold tumors into hot tumors, thereby boosting the effectiveness of immunotherapy. Despite extensive research on hypoxia and breast cancer, few studies systematically link hypoxia with breast cancer cold/hot tumor phenotypes and immunotherapy. This review aims to explore the interconnections between hypoxia, tumor immune phenotypes, and immunotherapy in breast cancer, offering an integrative perspective that could inform strategies to enhance immune responsiveness. By examining current evidence, the review provides a theoretical framework for future studies focused on converting cold tumors into hot tumors in the context of breast cancer. However, current technologies for improving tumor hypoxic microenvironments remain limited. It is hoped that more effective methods will be developed in the future to improve tumor hypoxia, leading to new therapeutic approaches for breast cancer and potentially other cancers. At the same time, improving hypoxia in breast cancer may lead to side effects such as tumor growth promotion, drug resistance, metabolic reprogramming, and immune evasion. Therefore, careful consideration is necessary for clinical applications.

## Data Availability

Data are available upon reasonable request to the corresponding author.

## Materials availability

Materials are available upon reasonable request to the corresponding author.

## References

[1] Chang PH, Chen MC, Tsai YP, Tan GYT, Hsu PH, Jeng YM, et al. Interplay between desmoglein2 and hypoxia controls metastasis in breast cancer. Proc Natl Acad Sci USA. 2021;118:e2014408118. 10.1073/pnas.2014408118 PMC7826351 33431674

[2] Semenza GL. The hypoxic tumor microenvironment: a driving force for breast cancer progression. Biochim Biophys Acta. 2016;1863:382–91. 26079100 10.1016/j.bbamcr.2015.05.036 PMC4678039

[3] Ray SK, Mukherjee S. Hyperoxic-hypoxic paradox: breast cancer microenvironment and an innovative treatment strategy. Anticancer Agents Med Chem. 2024;24:729–32. 38415470 10.2174/0118715206290816240220062545

[4] Movahedi K, Laoui D, Gysemans C, Baeten M, Stangé G, Van den Bossche J, et al. Different tumor microenvironments contain functionally distinct subsets of macrophages derived from Ly6C(high) monocytes. Cancer Res. 2010;70:5728–39. 20570887 10.1158/0008-5472.CAN-09-4672

[5] Chen Z, Han F, Du Y, Shi H, Zhou W. Hypoxic microenvironment in cancer: molecular mechanisms and therapeutic interventions. Signal Transduct Target Ther. 2023;8:70. 36797231 10.1038/s41392-023-01332-8 PMC9935926

[6] Ren X, Guo S, Guan X, Kang Y, Liu J, Yang X. Immunological classification of tumor types and advances in precision combination immunotherapy. Front Immunol. 2022;13:790113. 35296094 10.3389/fimmu.2022.790113 PMC8918549

[7] Kwan A, Winder N, Muthana M. Oncolytic virotherapy treatment of breast cancer: barriers and recent advances. Viruses. 2021;13:1128. 10.3390/v13061128 PMC8230950 34208264

[8] Semenza GL. Hypoxia-inducible factors: mediators of cancer progression and targets for cancer therapy. Trends Pharm Sci. 2012;33:207–14. 22398146 10.1016/j.tips.2012.01.005 PMC3437546

[9] Wenger RH. Cellular adaptation to hypoxia: O2-sensing protein hydroxylases, hypoxia-inducible transcription factors, and O2-regulated gene expression. FASEB J. 2002;16:1151–62. 12153983 10.1096/fj.01-0944rev

[10] Yang R, Chen H, Xing L, Wang B, Hu M, Ou X, et al. Hypoxia-induced circWSB1 promotes breast cancer progression through destabilizing p53 by interacting with USP10. Mol Cancer. 2022;21:88. 35351136 10.1186/s12943-022-01567-z PMC8961958

[11] Andrysik Z, Bender H, Galbraith MD, Espinosa JM. Multi-omics analysis reveals contextual tumor suppressive and oncogenic gene modules within the acute hypoxic response. Nat Commun. 2021;12:1375. 33654095 10.1038/s41467-021-21687-2 PMC7925689

[12] Recchia AG, De Francesco EM, Vivacqua A, Sisci D, Panno ML, Andò S, et al. The G protein-coupled receptor 30 is up-regulated by hypoxia-inducible factor-1alpha (HIF-1alpha) in breast cancer cells and cardiomyocytes. J Biol Chem. 2011;286:10773–82. 21266576 10.1074/jbc.M110.172247 PMC3060528

[13] Avena P, Casaburi I, Zavaglia L, Nocito MC, La Padula D, Rago V, et al. 27-hydroxycholesterol binds GPER and induces progression of estrogen receptor-negative breast cancer. Cancers. 2022;14:1521. 10.3390/cancers14061521 PMC8946696 35326671

[14] de Heer EC, Jalving M, Harris AL. HIFs, angiogenesis, and metabolism: elusive enemies in breast cancer. J Clin Invest. 2020;130:5074–87. 32870818 10.1172/JCI137552 PMC7524491

[15] Wang R, Godet I, Yang Y, Salman S, Lu H, Lyu Y, et al. Hypoxia-inducible factor-dependent ADAM12 expression mediates breast cancer invasion and metastasis. Proc Natl Acad Sci USA. 2021;118:e2020490118. 10.1073/pnas.2020490118 PMC8126789 33952697

[16] Dhamdhere SG, Bansal A, Singh P, Kakani P, Agrawal S, Samaiya A, et al. Hypoxia-induced ATF3 escalates breast cancer invasion by increasing collagen deposition via P4HA1. Cell Death Dis. 2025;16:142. 40016181 10.1038/s41419-025-07461-y PMC11868403

[17] Moon EJ, Mello SS, Li CG, Chi JT, Thakkar K, Kirkland JG, et al. The HIF target MAFF promotes tumor invasion and metastasis through IL11 and STAT3 signaling. Nat Commun. 2021;12:4308. 34262028 10.1038/s41467-021-24631-6 PMC8280233

[18] Shan Y, You B, Shi S, Shi W, Zhang Z, Zhang Q, et al. Hypoxia-induced matrix metalloproteinase-13 expression in exosomes from nasopharyngeal carcinoma enhances metastases. Cell Death Dis. 2018;9:382. 29515112 10.1038/s41419-018-0425-0 PMC5841433

[19] Xiang L, Semenza GL. Hypoxia-inducible factors promote breast cancer stem cell specification and maintenance in response to hypoxia or cytotoxic chemotherapy. Adv Cancer Res. 2019;141:175–212. 30691683 10.1016/bs.acr.2018.11.001

[20] Bradley CA, Salto-Tellez M, Laurent-Puig P, Bardelli A, Rolfo C, Tabernero J, et al. Targeting c-MET in gastrointestinal tumours: rationale, opportunities and challenges. Nat Rev Clin Oncol. 2017;14:562–76. 28374784 10.1038/nrclinonc.2017.40

[21] Yang Y, Chen C, Zuo Q, Lu H, Salman S, Lyu Y, et al. NARF is a hypoxia-induced coactivator for OCT4-mediated breast cancer stem cell specification. Sci Adv. 2022;8:eabo5000. 36490339 10.1126/sciadv.abo5000 PMC9733926

[22] Yan Y, He M, Zhao L, Wu H, Zhao Y, Han L, et al. A novel HIF-2α targeted inhibitor suppresses hypoxia-induced breast cancer stemness via SOD2-mtROS-PDI/GPR78-UPR(ER) axis. Cell Death Differ. 2022;29:1769–89. 35301432 10.1038/s41418-022-00963-8 PMC9433403

[23] Kocianova E, Piatrikova V, Golias T. Revisiting the Warburg effect with focus on lactate. Cancers. 2022;14:6028. 10.3390/cancers14246028 PMC9776395 36551514

[24] Gao FY, Li XT, Xu K, Wang RT, Guan XX. c-MYC mediates the crosstalk between breast cancer cells and tumor microenvironment. Cell Commun Signal. 2023;21:28. 36721232 10.1186/s12964-023-01043-1 PMC9887805

[25] Dhanasekaran R, Deutzmann A, Mahauad-Fernandez WD, Hansen AS, Gouw AM, Felsher DW. The MYC oncogene - the grand orchestrator of cancer growth and immune evasion. Nat Rev Clin Oncol. 2022;19:23–36. 34508258 10.1038/s41571-021-00549-2 PMC9083341

[26] Li Y, Sun XX, Qian DZ, Dai MS. Molecular crosstalk between MYC and HIF in cancer. Front Cell Dev Biol. 2020;8:590576. 33251216 10.3389/fcell.2020.590576 PMC7676913

[27] Singh D, Arora R, Kaur P, Singh B, Mannan R, Arora S. Overexpression of hypoxia-inducible factor and metabolic pathways: possible targets of cancer. Cell Biosci. 2017;7:62. 29158891 10.1186/s13578-017-0190-2 PMC5683220

[28] Göttgens EL, van den Heuvel CN, de Jong MC, Kaanders JH, Leenders WP, Ansems M, et al. ACLY (ATP Citrate Lyase) mediates radioresistance in head and neck squamous cell carcinomas and is a novel predictive radiotherapy biomarker. Cancers. 2019;11:1971. 10.3390/cancers11121971 PMC6966650 31817870

[29] Morotti M, Bridges E, Valli A, Choudhry H, Sheldon H, Wigfield S, et al. Hypoxia-induced switch in SNAT2/SLC38A2 regulation generates endocrine resistance in breast cancer. Proc Natl Acad Sci USA. 2019;116:12452–61. 31152137 10.1073/pnas.1818521116 PMC6589752

[30] Altman BJ, Stine ZE, Dang CV. From Krebs to clinic: glutamine metabolism to cancer therapy. Nat Rev Cancer. 2016;16:619–34. 27492215 10.1038/nrc.2016.71 PMC5484415

[31] Infantino V, Santarsiero A, Convertini P, Todisco S, Iacobazzi V. Cancer cell metabolism in hypoxia: role of HIF-1 as key regulator and therapeutic target. Int J Mol Sci. 2021;22:5703. 10.3390/ijms22115703 PMC8199012 34071836

[32] Ma S, Zhao Y, Lee WC, Ong LT, Lee PL, Jiang Z, et al. Hypoxia induces HIF1α-dependent epigenetic vulnerability in triple negative breast cancer to confer immune effector dysfunction and resistance to anti-PD-1 immunotherapy. Nat Commun. 2022;13:4118. 35840558 10.1038/s41467-022-31764-9 PMC9287350

[33] Di Carlo SE, Raffenne J, Varet H, Ode A, Granados DC, Stein M, et al. Depletion of slow-cycling PDGFRα(+)ADAM12(+) mesenchymal cells promotes antitumor immunity by restricting macrophage efferocytosis. Nat Immunol. 2023;24:1867–78. 37798557 10.1038/s41590-023-01642-7 PMC10602852

[34] Ivashkiv LB. The hypoxia-lactate axis tempers inflammation. Nat Rev Immunol. 2020;20:85–6. 31819164 10.1038/s41577-019-0259-8 PMC7021227

[35] Allard B, Allard D, Buisseret L, Stagg J. The adenosine pathway in immuno-oncology. Nat Rev Clin Oncol. 2020;17:611–29. 32514148 10.1038/s41571-020-0382-2

[36] Antonioli L, Blandizzi C, Pacher P, Haskó G. Immunity, inflammation and cancer: a leading role for adenosine. Nat Rev Cancer. 2013;13:842–57. 24226193 10.1038/nrc3613

[37] Sek K, Mølck C, Stewart GD, Kats L, Darcy PK, Beavis PA. Targeting adenosine receptor signaling in cancer immunotherapy. Int J Mol Sci. 2018;19:3837. 10.3390/ijms19123837 PMC6321150 30513816

[38] Vigano S, Alatzoglou D, Irving M, Ménétrier-Caux C, Caux C, Romero P, et al. Targeting adenosine in cancer immunotherapy to enhance T-cell function. Front Immunol. 2019;10:925. 31244820 10.3389/fimmu.2019.00925 PMC6562565

[39] Noman MZ, Janji B, Hu S, Wu JC, Martelli F, Bronte V, et al. Tumor-promoting effects of myeloid-derived suppressor cells are potentiated by hypoxia-induced expression of miR-210. Cancer Res. 2015;75:3771–87. 26206559 10.1158/0008-5472.CAN-15-0405

[40] Burrows N, Bashford-Rogers RJM, Bhute VJ, Peñalver A, Ferdinand JR, Stewart BJ, et al. Dynamic regulation of hypoxia-inducible factor-1α activity is essential for normal B cell development. Nat Immunol. 2020;21:1408–20. 32868930 10.1038/s41590-020-0772-8 PMC7613233

[41] Sami E, Paul BT, Koziol JA, ElShamy WM. The immunosuppressive microenvironment in BRCA1-IRIS-overexpressing TNBC tumors is induced by bidirectional interaction with tumor-associated macrophages. Cancer Res. 2020;80:1102–17. 31911557 10.1158/0008-5472.CAN-19-2374 PMC7056552

[42] Bai R, Li Y, Jian L, Yang Y, Zhao L, Wei M. The hypoxia-driven crosstalk between tumor and tumor-associated macrophages: mechanisms and clinical treatment strategies. Mol Cancer. 2022;21:177. 36071472 10.1186/s12943-022-01645-2 PMC9454207

[43] Li Q, Liu M, Sun Y, Jin T, Zhu P, Wan X, et al. SLC6A8-mediated intracellular creatine accumulation enhances hypoxic breast cancer cell survival via ameliorating oxidative stress. J Exp Clin Cancer Res. 2021;40:168. 33990217 10.1186/s13046-021-01933-7 PMC8120850

[44] Ji L, Zhao X, Zhang B, Kang L, Song W, Zhao B, et al. Slc6a8-mediated creatine uptake and accumulation reprogram macrophage polarization via regulating cytokine responses. Immunity. 2019;51:272–84.e7. 31399282 10.1016/j.immuni.2019.06.007

[45] Rønnov-Jessen L, Petersen OW, Koteliansky VE, Bissell MJ. The origin of the myofibroblasts in breast cancer. Recapitulation of tumor environment in culture unravels diversity and implicates converted fibroblasts and recruited smooth muscle cells. J Clin Invest. 1995;95:859–73. 7532191 10.1172/JCI117736 PMC295570

[46] Sun K, Tang S, Hou Y, Xi L, Chen Y, Yin J, et al. Oxidized ATM-mediated glycolysis enhancement in breast cancer-associated fibroblasts contributes to tumor invasion through lactate as metabolic coupling. eBioMedicine. 2019;41:370–83. 30799198 10.1016/j.ebiom.2019.02.025 PMC6442874

[47] De Francesco EM, Lappano R, Santolla MF, Marsico S, Caruso A, Maggiolini M. HIF-1α/GPER signaling mediates the expression of VEGF induced by hypoxia in breast cancer associated fibroblasts (CAFs). Breast Cancer Res. 2013;15:R64. 23947803 10.1186/bcr3458 PMC3978922

[48] Willson JA, Arienti S, Sadiku P, Reyes L, Coelho P, Morrison T, et al. Neutrophil HIF-1α stabilization is augmented by mitochondrial ROS produced via the glycerol 3-phosphate shuttle. Blood. 2022;139:281–6. 34411229 10.1182/blood.2021011010 PMC8832465

[49] Jiang J, Wang GZ, Wang Y, Huang HZ, Li WT, Qu XD. Hypoxia-induced HMGB1 expression of HCC promotes tumor invasiveness and metastasis via regulating macrophage-derived IL-6. Exp Cell Res. 2018;367:81–8. 29571949 10.1016/j.yexcr.2018.03.025

[50] Wang Z, Yang C, Li L, Jin X, Zhang Z, Zheng H, et al. Tumor-derived HMGB1 induces CD62L(dim) neutrophil polarization and promotes lung metastasis in triple-negative breast cancer. Oncogenesis. 2020;9:82. 32943604 10.1038/s41389-020-00267-x PMC7499196

[51] Naik A, Monjazeb AM, Decock J. The obesity paradox in cancer, tumor immunology, and immunotherapy: potential therapeutic implications in triple negative breast cancer. Front Immunol. 2019;10:1940. 31475003 10.3389/fimmu.2019.01940 PMC6703078

[52] Corzo CA, Condamine T, Lu L, Cotter MJ, Youn JI, Cheng P, et al. HIF-1α regulates function and differentiation of myeloid-derived suppressor cells in the tumor microenvironment. J Exp Med. 2010;207:2439–53. 20876310 10.1084/jem.20100587 PMC2964584

[53] Riera-Domingo C, Audigé A, Granja S, Cheng WC, Ho PC, Baltazar F, et al. Immunity, hypoxia, and metabolism-the Ménage à trois of cancer: implications for immunotherapy. Physiol Rev. 2020;100:1–102. 31414610 10.1152/physrev.00018.2019

[54] Winning S, Fandrey J. Dendritic cells under hypoxia: how oxygen shortage affects the linkage between innate and adaptive immunity. J Immunol Res. 2016;2016:5134329. 26966693 10.1155/2016/5134329 PMC4757696

[55] Ogino T, Onishi H, Suzuki H, Morisaki T, Tanaka M, Katano M. Inclusive estimation of complex antigen presentation functions of monocyte-derived dendritic cells differentiated under normoxia and hypoxia conditions. Cancer Immunol Immunother. 2012;61:409–24. 21932134 10.1007/s00262-011-1112-5 PMC11029581

[56] Chen DS, Mellman I. Elements of cancer immunity and the cancer-immune set point. Nature. 2017;541:321–30. 28102259 10.1038/nature21349

[57] Hegde PS, Karanikas V, Evers S. The where, the when, and the how of immune monitoring for cancer immunotherapies in the era of checkpoint inhibition. Clin Cancer Res. 2016;22:1865–74. 27084740 10.1158/1078-0432.CCR-15-1507

[58] Liu YT, Sun ZJ. Turning cold tumors into hot tumors by improving T-cell infiltration. Theranostics. 2021;11:5365–86. 33859752 10.7150/thno.58390 PMC8039952

[59] Hegde PS, Chen DS. Top 10 challenges in cancer immunotherapy. Immunity. 2020;52:17–35. 31940268 10.1016/j.immuni.2019.12.011

[60] DeVito NC, Plebanek MP, Theivanthiran B, Hanks BA. Role of tumor-mediated dendritic cell tolerization in immune evasion. Front Immunol. 2019;10:2876. 31921140 10.3389/fimmu.2019.02876 PMC6914818

[61] Hsu J, Hodgins JJ, Marathe M, Nicolai CJ, Bourgeois-Daigneault MC, Trevino TN, et al. Contribution of NK cells to immunotherapy mediated by PD-1/PD-L1 blockade. J Clin Invest. 2018;128:4654–68. 30198904 10.1172/JCI99317 PMC6159991

[62] Zhang J, Huang D, Saw PE, Song E. Turning cold tumors hot: from molecular mechanisms to clinical applications. Trends Immunol. 2022;43:523–45. 35624021 10.1016/j.it.2022.04.010

[63] Chen DS, Mellman I. Oncology meets immunology: the cancer-immunity cycle. Immunity. 2013;39:1–10. 23890059 10.1016/j.immuni.2013.07.012

[64] Spranger S, Bao R, Gajewski TF. Melanoma-intrinsic β-catenin signalling prevents anti-tumour immunity. Nature. 2015;523:231–5. 25970248 10.1038/nature14404

[65] Peng W, Chen JQ, Liu C, Malu S, Creasy C, Tetzlaff MT, et al. Loss of PTEN promotes resistance to T cell-mediated immunotherapy. Cancer Discov. 2016;6:202–16. 26645196 10.1158/2159-8290.CD-15-0283 PMC4744499

[66] Hamarsheh S, Groß O, Brummer T, Zeiser R. Immune modulatory effects of oncogenic KRAS in cancer. Nat Commun. 2020;11:5439. 33116132 10.1038/s41467-020-19288-6 PMC7595113

[67] Kortlever RM, Sodir NM, Wilson CH, Burkhart DL, Pellegrinet L, Brown Swigart L, et al. Myc cooperates with ras by programming inflammation and immune suppression. Cell. 2017;171:1301–15.e14. 29195074 10.1016/j.cell.2017.11.013 PMC5720393

[68] Nuhn L, De Koker S, Van Lint S, Zhong Z, Catani JP, Combes F, et al. Nanoparticle-conjugate TLR7/8 agonist localized immunotherapy provokes safe antitumoral responses. Adv Mater. 2018;30:e1803397. 30276880 10.1002/adma.201803397

[69] Russell L, Peng KW, Russell SJ, Diaz RM. Oncolytic viruses: priming time for cancer immunotherapy. BioDrugs. 2019;33:485–501. 31321623 10.1007/s40259-019-00367-0 PMC6790338

[70] Twumasi-Boateng K, Pettigrew JL, Kwok YYE, Bell JC, Nelson BH. Oncolytic viruses as engineering platforms for combination immunotherapy. Nat Rev Cancer. 2018;18:419–32. 29695749 10.1038/s41568-018-0009-4

[71] van der Burg SH, Arens R, Ossendorp F, van Hall T, Melief CJ. Vaccines for established cancer: overcoming the challenges posed by immune evasion. Nat Rev Cancer. 2016;16:219–33. 26965076 10.1038/nrc.2016.16

[72] Wu Y, Yi Z, Li J, Wei Y, Feng R, Liu J, et al. FGFR blockade boosts T cell infiltration into triple-negative breast cancer by regulating cancer-associated fibroblasts. Theranostics. 2022;12:4564–80. 35832090 10.7150/thno.68972 PMC9254240

[73] Kirchhammer N, Trefny MP, Natoli M, Brücher D, Smith SN, Werner F, et al. NK cells with tissue-resident traits shape response to immunotherapy by inducing adaptive antitumor immunity. Sci Transl Med. 2022;14:eabm9043. 35857639 10.1126/scitranslmed.abm9043

[74] Garris CS, Arlauckas SP, Kohler RH, Trefny MP, Garren S, Piot C, et al. Successful anti-PD-1 cancer immunotherapy requires T cell-dendritic cell crosstalk involving the cytokines IFN-γ and IL-12. Immunity. 2018;49:1148–61.e7. 30552023 10.1016/j.immuni.2018.09.024 PMC6301092

[75] Tugues S, Burkhard SH, Ohs I, Vrohlings M, Nussbaum K, Vom Berg J, et al. New insights into IL-12-mediated tumor suppression. Cell Death Differ. 2015;22:237–46. 25190142 10.1038/cdd.2014.134 PMC4291488

[76] Chambers AM, Lupo KB, Matosevic S. Tumor microenvironment-induced immunometabolic reprogramming of natural killer cells. Front Immunol. 2018;9:2517. 30467503 10.3389/fimmu.2018.02517 PMC6235907

[77] Young A, Ngiow SF, Gao Y, Patch AM, Barkauskas DS, Messaoudene M, et al. A2AR adenosine signaling suppresses natural killer cell maturation in the tumor microenvironment. Cancer Res. 2018;78:1003–16. 29229601 10.1158/0008-5472.CAN-17-2826

[78] Jiang X, Wang J, Deng X, Xiong F, Zhang S, Gong Z, et al. The role of microenvironment in tumor angiogenesis. J Exp Clin Cancer Res. 2020;39:204. 32993787 10.1186/s13046-020-01709-5 PMC7526376

[79] Facciabene A, Peng X, Hagemann IS, Balint K, Barchetti A, Wang LP, et al. Tumour hypoxia promotes tolerance and angiogenesis via CCL28 and T(reg) cells. Nature. 2011;475:226–30. 21753853 10.1038/nature10169

[80] Hatfield SM, Kjaergaard J, Lukashev D, Belikoff B, Schreiber TH, Sethumadhavan S, et al. Systemic oxygenation weakens the hypoxia and hypoxia inducible factor 1α-dependent and extracellular adenosine-mediated tumor protection. J Mol Med. 2014;92:1283–92. 25120128 10.1007/s00109-014-1189-3 PMC4247798

[81] Hatfield SM, Kjaergaard J, Lukashev D, Schreiber TH, Belikoff B, Abbott R, et al. Immunological mechanisms of the antitumor effects of supplemental oxygenation. Sci Transl Med. 2015;7:277ra30. 25739764 10.1126/scitranslmed.aaa1260 PMC4641038

[82] Kaneda MM, Messer KS, Ralainirina N, Li H, Leem CJ, Gorjestani S, et al. PI3Kγ is a molecular switch that controls immune suppression. Nature. 2016;539:437–42. 27642729 10.1038/nature19834 PMC5479689

[83] Damgaci S, Ibrahim-Hashim A, Enriquez-Navas PM, Pilon-Thomas S, Guvenis A, Gillies RJ. Hypoxia and acidosis: immune suppressors and therapeutic targets. Immunology. 2018;154:354–62. 29485185 10.1111/imm.12917 PMC6002221

[84] Noman MZ, Desantis G, Janji B, Hasmim M, Karray S, Dessen P, et al. PD-L1 is a novel direct target of HIF-1α, and its blockade under hypoxia enhanced MDSC-mediated T cell activation. J Exp Med. 2014;211:781–90. 24778419 10.1084/jem.20131916 PMC4010891

[85] Pan WL, Tan Y, Meng W, Huang NH, Zhao YB, Yu ZQ, et al. Microenvironment-driven sequential ferroptosis, photodynamic therapy, and chemotherapy for targeted breast cancer therapy by a cancer-cell-membrane-coated nanoscale metal-organic framework. Biomaterials. 2022;283:121449. 35247637 10.1016/j.biomaterials.2022.121449

[86] Wu M, Chen T, Wang L, Akakuru OU, Ma X, Xu J, et al. The strategy of precise targeting and in situ oxygenating for enhanced triple-negative breast cancer chemophototherapy. Nanoscale. 2022;14:8349–61. 35635070 10.1039/d2nr00985d

[87] Gao P, Zhang H, Dinavahi R, Li F, Xiang Y, Raman V, et al. HIF-dependent antitumorigenic effect of antioxidants in vivo. Cancer Cell. 2007;12:230–8. 17785204 10.1016/j.ccr.2007.08.004 PMC2084208

[88] Luo S, Jiang Y, Anfu Z, Zhao Y, Wu X, Li M, et al. Targeting hypoxia-inducible factors for breast cancer therapy: a narrative review. Front Pharm. 2022;13:1064661. 10.3389/fphar.2022.1064661 PMC9751339 36532768

[89] Liu M, Liang Y, Zhu Z, Wang J, Cheng X, Cheng J, et al. Discovery of novel aryl carboxamide derivatives as hypoxia-inducible factor 1α signaling inhibitors with potent activities of anticancer metastasis. J Med Chem. 2019;62:9299–314. 31556611 10.1021/acs.jmedchem.9b01313

[90] Li G, Shao Y, Pan Y, Li Y, Wang Y, Wang L, et al. Total synthesis and biological evaluation of 7-hydroxyneolamellarin A as hypoxia-inducible factor-1α inhibitor for cancer therapy. Bioorg Med Chem Lett. 2021;50:128338. 34469710 10.1016/j.bmcl.2021.128338

[91] Corry PM, Dewhirst MW. Thermal medicine, heat shock proteins and cancer. Int J Hyperth. 2005;21:675–7. 10.1080/02656730500272856 16338847

[92] Dewhirst MW, Mowery YM, Mitchell JB, Cherukuri MK, Secomb TW. Rationale for hypoxia assessment and amelioration for precision therapy and immunotherapy studies. J Clin Invest. 2019;129:489–91. 30614815 10.1172/JCI126044 PMC6355230

[93] Brizel DM, Lin S, Johnson JL, Brooks J, Dewhirst MW, Piantadosi CA. The mechanisms by which hyperbaric oxygen and carbogen improve tumour oxygenation. Br J Cancer. 1995;72:1120–4. 7577456 10.1038/bjc.1995.474 PMC2033965

[94] Dewhirst MW, Secomb TW. Transport of drugs from blood vessels to tumour tissue. Nat Rev Cancer. 2017;17:738–50. 29123246 10.1038/nrc.2017.93 PMC6371795

[95] Schadler KL, Thomas NJ, Galie PA, Bhang DH, Roby KC, Addai P, et al. Tumor vessel normalization after aerobic exercise enhances chemotherapeutic efficacy. Oncotarget. 2016;7:65429–40. 27589843 10.18632/oncotarget.11748 PMC5323166

[96] Lai C, Luo B, Shen J, Shao J. Biomedical engineered nanomaterials to alleviate tumor hypoxia for enhanced photodynamic therapy. Pharmacol Res. 2022;186:106551. 36370918 10.1016/j.phrs.2022.106551

[97] Zhou F, Fu T, Huang Q, Kuai H, Mo L, Liu H, et al. Hypoxia-activated PEGylated conditional aptamer/antibody for cancer imaging with improved specificity. J Am Chem Soc. 2019;141:18421–7. 31584808 10.1021/jacs.9b05063

[98] Li X, Wu Y, Zhang R, Bai W, Ye T, Wang S. Oxygen-based nanocarriers to modulate tumor hypoxia for ameliorated anti-tumor therapy: fabrications, properties, and future directions. Front Mol Biosci. 2021;8:683519. 34277702 10.3389/fmolb.2021.683519 PMC8281198

[99] Zuo H, Tao J, Shi H, He J, Zhou Z, Zhang C. Platelet-mimicking nanoparticles co-loaded with W(18)O(49) and metformin alleviate tumor hypoxia for enhanced photodynamic therapy and photothermal therapy. Acta Biomater. 2018;80:296–307. 30223092 10.1016/j.actbio.2018.09.017

